# Relative kinetic expressions defining cleavage synchronicity are better predictors of blastocyst formation and quality than absolute time points

**DOI:** 10.1007/s10815-014-0341-x

**Published:** 2014-11-05

**Authors:** Murat Cetinkaya, Caroline Pirkevi, Hakan Yelke, Yesim Kumtepe Colakoglu, Zafer Atayurt, Semra Kahraman

**Affiliations:** Istanbul Memorial Hospital, Assisted Reproductive Techniques and Reproductive Genetics Center, Piyale Pasa Bulvari, 34385 Okmeydani, Sisli Istanbul/Turkey

**Keywords:** Time-lapse imaging, Morphokinetics, Synchronicity, Cleavage, Blastocyst

## Abstract

**Purpose:**

Morphology alone is not enough for the selection of the embryo (s) with the highest implantation potential and time-lapse imaging has added embryo development kinetics as another selection criterion. Therefore, a combination of morphology with kinetics has inspired a new field termed “morphokinetics”, providing a new way of evaluating and selecting embryos. The aim of the study was to identify a criterion *solely* based on morphokinetic data and available up to the 8-cell stage (t8) to predict blastocyst formation and quality.

**Methods:**

The study included 3,354 embryos, with annotations up to t8, and cultured until day 5 from 626 infertile patients. A total of 17 kinetic expressions, either absolute cleavage timings and time intervals or time ratios were tested retrospectively for the prediction of blastocyst formation and quality.

**Results:**

Relative timings (t8-t5, the cleavage synchronicity from 4 to 8 cells and from 2 to 8 cells) were found to be better indicators of blastocyst formation and quality when compared to absolute time-points. Especially, the cleavage synchronicity from 2 to 8 cells (CS2-8) = ((t3-t2) + (t5-t4))/(t8-t2)) was found to be the best predictor available on day 3 for blastocyst formation and quality (AUC:0.786; sensitivity: 83.43; specificity: 62.46).

**Conclusions:**

Time intervals and relative ratios based on selected cleavage cycles defining synchronicity allowed a specific analysis providing high predictivity of blastocyst formation and quality.

**Electronic supplementary material:**

The online version of this article (doi:10.1007/s10815-014-0341-x) contains supplementary material, which is available to authorized users.

## Introduction

Successful implantation requires a competent embryo, a receptive endometrium and a synchronized mother-embryo crosstalk that is regulated by endocrine, paracrine and autocrine interactions [1]. Culturing embryos until day 5 to select the best-morphology blastocyst ensures higher implantation and lower miscarriage rates, either after fresh transfer or following vitrification [2–5]. However, the cumulative clinical pregnancy rate of cleavage stage embryo transfers is higher [6]. Many in vitro fertilization (IVF) laboratories worldwide transfer cleavage-stage embryo (s) due to suspected negative effect of prolonged culture, low number of available embryos, and/or to avoid transfer cancellations. Thus, being able to predict blastocyst formation and quality potential on day 3 is essential to perform successful cleavage-stage transfers.

Scoring embryos is fundamental for IVF laboratories, but still remains open to intra and interpersonal variability, despite efforts to find more objective means [7, 8]. Attempts to find complementary methods for morphological assessment have been revealed as not effective or reliable in a clinical setting, to be time-consuming or very expensive, and thus difficult to adopt in daily practice [9–12].

Continuous monitoring of embryo development from fertilization to the blastocyst stage by automated time-lapse imaging has raised the possibility of examining the timing of embryonic cell division [13–16]. In the classical morphological evaluation, the embryos have to be removed daily from the incubator for few minutes to allow a static observation under the microscope, whereas in time-lapse systems embryos can be continuously monitored without being disturbed. In addition, time-lapse allows the observation of exact timing of cell divisions, compaction and blastocyst formation, generation and absorption of fragments and multinucleation. Thus, blastocyst evaluation in conventional incubation systems is based on morphologic criteria only, e.g. blastocoel formation and expansion, inner cell mass quality, and trophectoderm quality, whereas in time-lapse instruments kinetic parameters can be evaluated as well as pre-transfer final morphology [2, 15, 17].

The safety of embryo culture in time-lapse vs. conventional incubation has been demonstrated in embryos from fresh oocytes derived from donors or infertile patients [18, 19]. Also, the retrospective analysis of morphokinetic data from 247 embryos incubated in time-lapse and transferred on day 3 allowed the establishment of a concept by which a hierarchical model relying on morphokinetics can predict embryo implantation [17]. In this study, the most predictive parameters are described as being, first, the time of division to 5 cells (t5), second, the time between division to 3 cells and subsequent division to 4 cells (s2) and, finally, the time between division to 2 cells and division to 3 cells (cc2). In a following study, this hierarchical model has been used in an effort to correlate the kinetics of early embryo development with blastocyst formation and quality [20].

Dal Canto and colleagues have also investigated morphokinetics in the development to blastocyst and implantation [21]. Cleavage times to the 7- and 8-cell stages and relative intervals from the 4- to 8-cell stage, and also from the 5- to 8-cell stage, have been found to be statistically different for embryos arresting after the 8-cell stage, and for embryos developing to blastocysts. The ability of blastocysts to expand correlates with all cleavage times from the 3-cell stage onwards. Moreover, implanted embryos achieve the 8-cell stage earlier than those that did not implant. Therefore, the study shows that cleavage from the 2- to 8-cell stage occurs progressively earlier in embryos with the ability to develop to blastocysts, to expand, and also to implant [21]. These observations also infer that using cleavage times until the 5-cell stage may not be enough in a different IVF laboratory setting to predict blastocyst formation potential and implantation, as short shifts in early cleavage timings end up with longer lags from the 5- to the 8-cell stage and are hence more prominent.

However, a model based on some cleavage time intervals up to the 4-cell stage (t3-t2: time between cytokinesis 1 and 2; t4-t3: time between cytokinesis 2 and 3) infers that when used with traditional day 3 morphology it may help embryologists to predict usable blastocyst outcomes and reduce the variability among embryologists [22]. Furthermore, a prospective study reported that only the duration of the first cytokinesis, the duration of the 3-cell stage and direct cleavage to 3-cells predict development to high-quality blastocyst with an area under curve (AUC) of 0.69 [23].

We present here the retrospective morphokinetic data of 3,354 embryos cultured until day 5, the analysis of their cleavage timings, time intervals and also ratios reflecting cleavage synchronicity up to the 8-cell stage and evaluate their potential to predict blastocyst formation and quality. We finally report unified criteria to express the cleavage synchronicity of embryonic cell divisions.

## Materials and methods

### Patients

This retrospective observational cohort study was conducted in a private referral IVF clinic from October 2011 to March 2014 and was drawn from a total of 5,153 metaphase II oocytes generated in 648 IVF treatment cycles from 626 patients with various infertility causes, and cultured until day 5. (Supplementary Table 1). All protocols were approved by the institutional review board and all patients gave their informed consent prior to their inclusion in the study. Preimplantation Genetic Diagnosis (PGD) and Preimplantation Genetic Screening (PGS) cycles were excluded from the study. All embryos were obtained after fertilization by intracytoplasmic sperm injection (ICSI) and were part of our standard ART program, and their development was recorded using time-lapse technology (EmbryoScope™ time-lapse system, Unisense Fertilitech, Aarhus, Denmark).

### Ovarian stimulation

Recombinant FSH (rFSH; Gonal-F®; Merck Serono, Switzerland) was used for ovarian stimulation starting from the second day of menses at a dose of 150–225 IU, and when the leading follicle reached a diameter of 12–13 mm, 0.25 mg GnRH antagonist (Cetrotide®; Merck Serono, Switzerland) was administered daily. When two or more follicles had attained a minimum mean diameter of 18 mm, follicular maturation was achieved by using 250 μg of r-hCG (Ovitrelle®; Merck Serono, Switzerland). Transvaginal ultrasound - guided oocyte retrieval was scheduled 36 h later.

### Oocyte retrieval, denudation and ICSI

Follicles were aspirated and the cumulus-oocyte complexes (COC) were washed in human tubal fluid (HTF) medium (Life Global®, Brussels, Belgium). The COCs were then incubated for 3.5 h at 6 % CO_2_, 5 % O_2_ and 37 °C before denudation, which was carried out by mechanical pipetting in 40 IU/ml of hyaluronidase in HTF (Life Global®, Brussels, Belgium). After denudation, the oocytes were allowed to incubate for an additional 30 min. Four hours after pick-up, ICSI was performed in an HTF medium with HEPES (Life Global®, Brussels, Belgium). ICSI was performed at x400 magnification using Olympus IX70 and Olympus IX71 inverted microscopes. Subsequently, the injected oocytes were placed in special culture slides pre-equilibrated at least 4 h in advance at 6 % CO_2_, 5 % O_2_ and 37 °C (EmbryoSlide® culture disc, Unisense Fertilitech, Aarhus, Denmark).

### Embryo culture and incubation

Each of the 12 individual wells of the EmbryoSlide® culture disc was filled with 25 μl of a single step culture medium (Life Global®, Brussels, Belgium), supplemented with 10 % plasmanate (Life Global®, Brussels, Belgium), and covered with an overlay of 1.5 ml paraffin oil (Life Global®, Brussels, Belgium). Following ICSI, injected oocytes positioned in the wells of the slide were placed in a time-lapse incubator (EmbryoScope™, Unisense Fertilitech, Aarhus, Denmark) and incubated at 6 % CO_2_, 5 % O_2_ and 37 °C for 5 days until embryo transfer. The culture medium was refreshed on the afternoon of day 3 by replacing the incubated slide with a new pre-equilibrated slide prepared as described above.

Image stacks were acquired at seven focal planes every 20 min and data were continuously transferred to an external computer, EmbryoViewer® workstation (Unisense Fertilitech, Aarhus, Denmark). Embryo development was annotated by one investigator and crosschecked by two other assessors.

Annotations of embryos incubated in the EmbryoScope™ were done according to a detailed Standard Operating Procedure (SOP) which was implemented in our IVF laboratory in order to prevent inter- and intra-observer variations. This SOP is often checked and amended regarding new articles and outcomes of our quality assurance program. The embryologists trained and allowed to annotate embryos in the EmbryoScope™ have to pass a test consisting in annotating pre-selected embryos cultured until day 5, every other month. Moreover, in line with the ISO 15189 accreditation program, embryologists are enrolled in web-based education and quality assurance schemes consisting in ranking and assessing oocytes, cleavage-stage embryos and blastocysts to minimize inter- and intra-observer variations.

### Time-lapse evaluation and embryo scoring

All relevant events (fertilization, cleavages, morula and blastocyst formation) were checked on a daily basis, and time of cleavage to 2-blastomere embryo t2, and subsequent divisions t3, t4, t5, t6, t7, t8 were recorded in the EmbryoViewer® workstation. The time of all mitotic events was expressed as hours post-ICSI. Blastocysts were scored according to Gardner’s classification (114–120 h post-ICSI), and classified into three groups: top quality (TQ), good quality (GQ) and bad quality (BQ) blastocysts. The TQ designation includes 3AA, 4AA and 5AA blastocysts, whereas GQ comprises those graded as 3/ 4/ 5BB, AB or BA. Blastocysts of inferior quality were designated as BQ blastocysts. Embryos manifesting a developmental arrest after the 8-cell stage were noted as AE.

### Equations defining time ratios

All data were recorded in the EmbryoViewer® workstation initially and exported for further analysis into Microsoft Excel. Spreadsheet analysis was performed for the cleavage timings from t2 to t8, six cleavage cycle intervals (t3-t2; t4-t3; t5-t4; t5-t3; t8-t5; t8-t2) and for four ratio derived from morphokinetic parameters:

- CS2-8: The Cleavage Synchronicity from the 2- to 8-cell stage (named also synchronicity of cleavage cycles) was defined and calculated by the formula:

CS2-8 = ((t3-t2) + (t5-t4)) / (t8-t2)

This formula reflects the ratio of time the embryo spends at the 2-cell stage and at the 4-cell stage over the time from the 2- to the 8-cell stage (Supplementary Fig. 1). Although each blastomere basically behaves independently during mitotic cell divisions, an embryonic synchronicity exists, such that uneven cell stages represent very short time frames during the 5 days of preimplantation embryo development that we are able to observe by time-lapse imaging. Therefore, the ideal ratio that an embryo could obtain for CS2-8 is close to 1, whereas in the worst case scenario the formula will have a value tending to 0.

- CS4-8: The Cleavage Synchronicity from the 4- to 8-cell stage was defined and calculated by the formula:

CS4-8 = (t8-t5) / (t8-t4)

This equation represents the ratio of time spent by an embryo from the 5 to the 8-cell stage, in relation to the time from the 4- to the 8-cell stage. In other words it reflects the ratio between time spent by embryos at the 5-, 6- and 7-cell stages when compared to the duration of the third cleavage cycle. The ideal ratio that an embryo could obtain for CS4-8 is close to 0, whereas in the worst case scenario the formula will have a value tending to 1.

- CS2-4: The Cleavage Synchronicity from the 2- to 4-cell stage was defined and calculated by the formula:

CS2-4 = (t4-t3) / (t4-t2)

This equation represents the ratio of time spent by an embryo from the 3 to the 4-cell stage, in relation to the time from the 2- to the 4-cell stage. In other words it reflects the ratio between time spent by embryos at the 3-cell stage when compared to the duration of the second cleavage cycle. The ideal ratio that an embryo could obtain for CS2-4 is close to 0, whereas in the worst case scenario the formula will have a value tending to 1.

- DR: The DNA Replication time ratio was defined and calculated by the formula:

DR = (t3-t2) / (t5-t3)

From the 2- to the 3-cell stage the embryo replicates the genomic material that will be needed until the 4-cell stage. Then, the second replication occurs during the 3- to the 5-cell stage and allows the accumulation of the DNA that will be used for the mitotic divisions until the 8-cell stage. Therefore, a ratio exists between these two events and was calculated by the formula above.

### Statistical analysis

Statistically significant differences in continuous variables were analyzed using the Mann–Whitney U test since the data did not follow a normal distribution (normality was assessed by Kolmogorov-Smirnov test). Statistical significance was determined as *p* < 0.05. The classification performance of each variable for blastocyst outcome and morphology was tested by calculating the area under curve (AUC) of Receiver Operating Characteristics (ROC). The data were analyzed in quartiles and for each variable the highest interquartile difference of TQ + GQ blastocysts rates was calculated in order to add additional insight into the classification. All statistical analyses were performed using the SPSS 16.0 statistical package (SPSS Inc., Chicago, IL, USA) and MedCalc Statistical Software version 13.2.0 (MedCalc Software bvba, Ostend, Belgium).

Embryos manifesting a developmental arrest before achieving eight cells (*n* = 1,006), or resulting in division by zero error for at least one of the formulas mentioned before (*n* = 123) were excluded from the study. Therefore, 3,354 embryos with a t8 annotation and reaching day 5 were subclassified as follows: 644 TQ and 1,197 GQ blastocysts, representing 54.9 % (*n* = 1,841) of the total cohort and 722 BQ blastocysts and 791 embryos with a developmental arrest after the 8 cell-stage, representing 45.1 % (*n* = 1,513) of the studied embryo set (Supplementary Fig. 2).

## Results

All of the cleavage timings, time intervals and time ratios were found to be significantly different between TQ + GQ and BQ + AE embryos except for t3 (Table 1). Thus, the performance to classify the blastocyst outcome and morphology of each variable was tested individually by a ROC analysis, as AUC values closer to 1 together with high sensitivity and specificity indicate a better classification power of the variable (Table 2). Then, the highest percent differences in the quartile distribution of TQ + GQ vs. BQ + AE embryos were compared (Supplementary Table 2). Cleavage timings, time intervals and time ratios are further described below in three distinct categories.

**Table 1:** Cleavage timings, time intervals and time ratios are compared between TQ + GQ and BQ + AE embryos

	TQ + GQ		BQ + AE		*p*
	Median	Interquartile Range	Median	Interquartile Range	
t2	26.00	24.11 to 27.96	27.63	25.11 to 30.55	<0.0001
t3	37.20	34.99 to 39.65	37.48	33.53 to 40.88	0.9788
t4	38.10	35.76 to 40.63	39.78	36.44 to 43.61	<0.0001
t5	50.34	47.06 to 53.72	49.06	42.49 to 55.04	<0.0001
t6	51.87	48.47 to 55.09	53.50	48.16 to 59.39	<0.0001
t7	53.51	49.89 to 57.20	57.22	51.81 to 64.16	<0.0001
t8	55.44	51.51 to 60.42	61.86	55.09 to 70.11	<0.0001
t3-t2	11.31	10.52 to 12.10	11.23	3.66 to 12.50	<0.0001
t4-t3	0.50	0.24 to 1.25	1.00	0.00 to 3.00	<0.0001
t5-t4	12.35	11.01 to 13.75	10.67	1.34 to 13.81	<0.0001
t5-t3	13.04	12.00 to 14.50	12.76	9.67 to 15.40	<0.0001
t8-t5	4.00	2.38 to 7.67	13.53	6.00 to 19.56	<0.0001
t8-t2	29.08	26.28 to 33.02	33.28	28.11 to 41.00	<0.0001
CS2-8	0.83	0.73 to 0.89	0.53	0.34 to 0.77	<0.0001
CS2-4	0.04	0.01 to 0.09	0.08	0.01 to 0.28	<0.0001
CS4-8	0.24	0.16 to 0.38	0.58	0.31 to 0.93	<0.0001
DR	0.85	0.78 to 0.93	0.80	0.44 to 0.98	<0.0001

**Table 2:** ROC curve analysis of the variables

	AUC (95 % CI)	Sensitivity (%)	Specificity (%)
t2	0.638 (0.621 to 0.654)	74.63	47.72
t3	0.500 (0.483 to 0.517)	92.45	20.82
t4	0.601 (0.584 to 0.618)	77.19	41.57
t5	0.555 (0.538 to 0.572)	91.09	29.81
t6	0.567 (0.550 to 0.583)	83.87	34.90
t7	0.646 (0.630 to 0.662)	83.65	42.70
t8	0.690 (0.674 to 0.706)	74.58	56.38
t3-t2	0.546 (0.529 to 0.563)	97.45	28.35
t4-t3	0.594 (0.578 to 0.611)	84.30	36.55
t5-t3	0.541 (0.524 to 0.558)	90.06	31.13
t5-t4	0.630 (0.613 to 0.646)	92.07	41.77
t8-t5	0.778 (0.763 to 0.792)	77.35	67.75
t8-t2	0.658 (0.642 to 0.674)	72.90	54.26
CS2-8	0.786 (0.772 to 0.800)	83.43	62.46
CS2-4	0.616 (0.599 to 0.633)	93.09	31.12
CS4-8	0.776 (0.761 to 0.790)	82.47	61.92
DR	0.579 (0.562 to 0.596)	94.44	32.89

### Cleavage timings

**Fig. 1:**
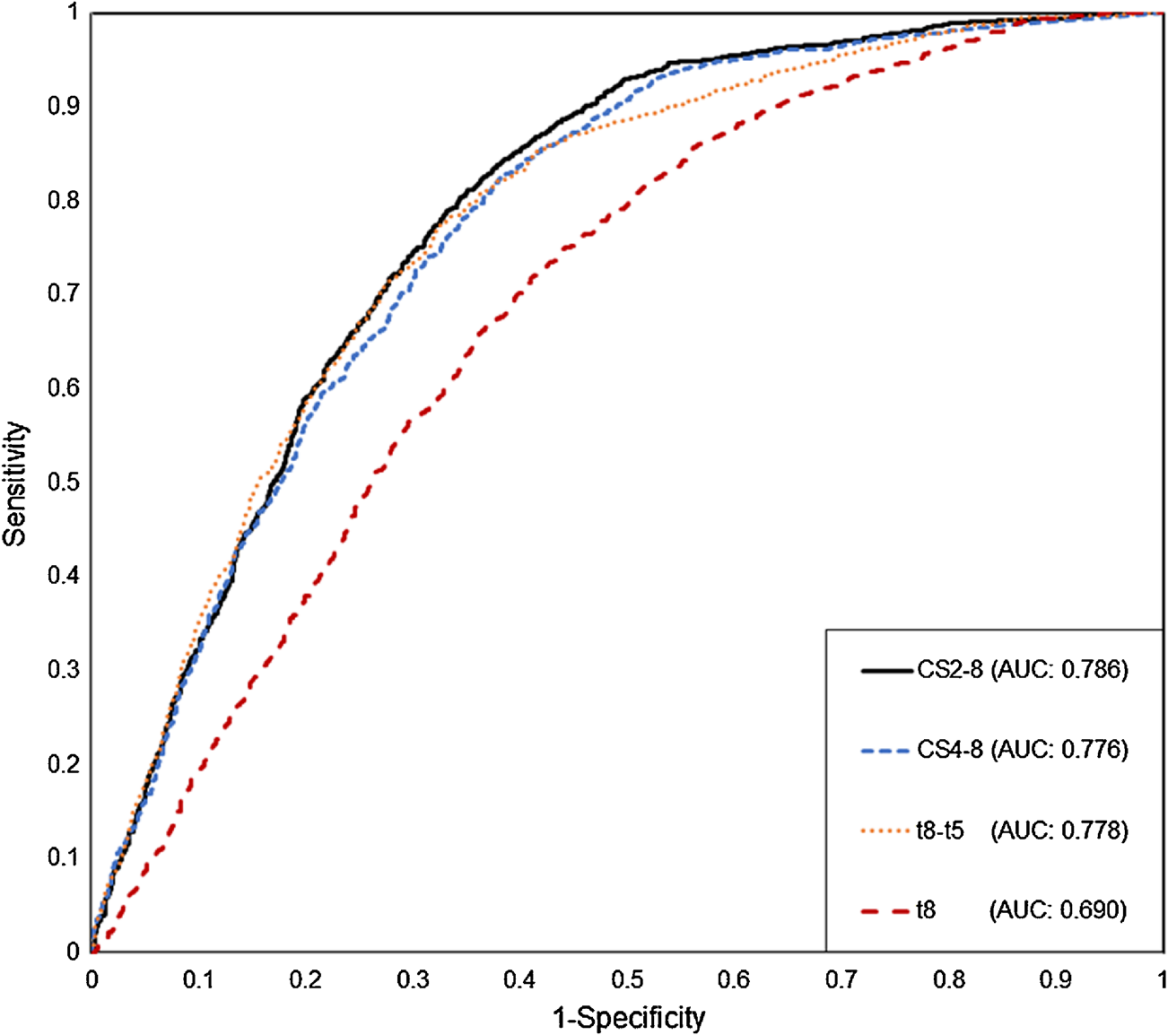
Comparison of ROC curves

The highest AUC was noted for t8 (AUC: 0.690; 95 % CI: 0.674 to 0.706; sensitivity: 74.58 %; specificity: 56.38 %) (Fig. 1; Table 2). When the t8 values were analyzed in quartiles, the chance of forming a usable blastocyst gradually decreased from 69.4 to 27.2 %, from the first to the fourth quartile, respectively (Supplementary Table 2). Although ROC analyses resulted in relatively low AUC values for absolute time points (<0.7), the quartile approach indicated that embryos reaching the t2 and t8 stages earlier (Q1 and Q2) have a higher chance of forming TQ or GQ blastocysts when compared to slower embryos (Q3 and Q4) (Table 2; Supplementary Table 2).

### Time intervals

The highest AUC was obtained for t8-t5 (AUC: 0.778; 95 % CI: 0.763 to 0.792; sensitivity: 77.35 %; specificity: 67.75 %) (Fig. 1; Table 2). Following the same trend as t8, for t8-t5 the ratio of usable blastocysts decreased from 80.8 to 20.4 % from the first to the fourth quartile, respectively (60.4 % interquartile difference) (Supplementary Table 2). Other time intervals reflecting duration of cell cycles and synchrony (eg. t3-t2; t4-t3; t5-t3 and t5-t4) were found to have less classification power when compared to t8–t5.

### Time ratios

**Fig. 2:**
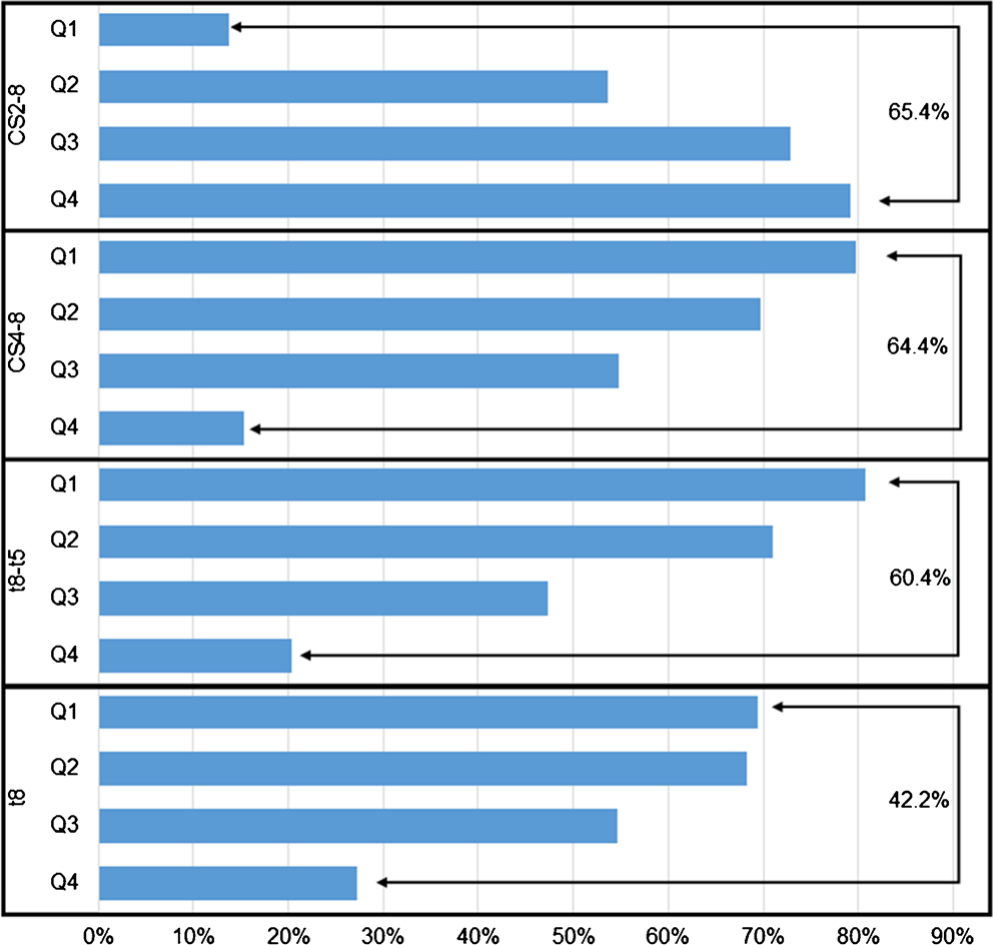
Distribution of TQ + GQ blastocysts among quartiles. The highest interquartile difference is shown on the right

Among all the tested variables, cleavage timings, time intervals and time ratios, the highest AUC was achieved for the expression defining cleavage synchronicity from 2 to 8 cells, CS2-8 (AUC: 0.786; 95 % CI: 0.772 to 0.800; sensitivity: 83.43 %; specificity: 62.46 %) (Fig. 1; Table 2). Moreover, the interquartile difference was the highest for CS2-8 (65.4 %): the ratio of TQ + GQ blastocysts located in the highest scored quartile (Q4) was 79.2 % and 13.8 % in the lowest scored quartile (Q1) (*p* < 0.0001) (Fig. 2; Supplementary Table 2).

The cleavage synchronicity from 4 to 8 cells, CS4-8 was observed as having the second highest AUC for the prediction of blastocyst formation and quality (AUC: 0.776; 95 % CI: 0.761 to 0.790; sensitivity: 82.47 %; specificity: 61.92 %) (Fig. 1). CS4-8 has also the second highest interquartile difference (64.4 %) (Fig. 2).

### Comparing blastocyst morphology with CS2-8 scores

**Fig. 3:**
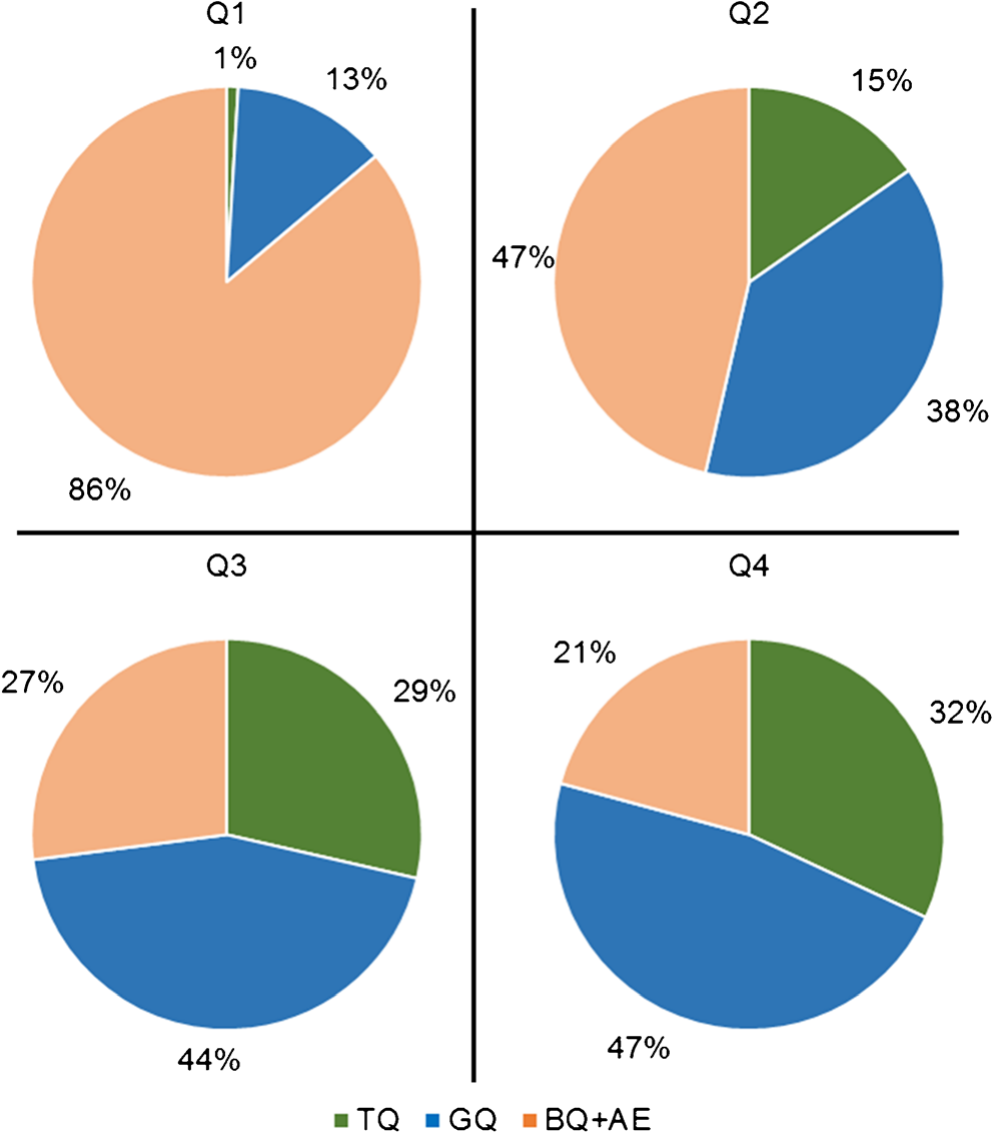
Distribution among quartiles of CS2-8 scores given on day 3 and comparison with the final morphology of the embryo on day 5

As the highest AUC and interquartile difference was obtained with CS2-8, a subanalysis was performed to compare retrospectively the predicted quality of blastocysts, calculated with annotations gathered until t8 and blinded to the quality of the blastocyst, with the day 5 morphology of embryos. Although 79.2 % of the embryos located in the fourth quartile were classified as usable blastocysts (32 % TQ and 47.2 % GQ), only 13.8 % of the embryos placed in the first quartile were of the same quality (1 % TQ and 12.9 % GQ). Therefore, 79.2 % and 72.9 % of the blastocysts were correctly predicted as being TQ or GQ blastocysts in the third and fourth quartiles, respectively (*p* < 0.0001). Also, the BQ + AE embryo rate was dramatically boosted to 86.2 % for the first quartile (Fig. 3). Likewise, the analysis of the 3,354 embryos by three morphology categories (TQ, GQ and BQ + AE) showed an increased rate of embryos scored on day 3 as belonging to the best or good group (Q3 + Q4 = 79 %) for the TQ blastocysts and 64 % for the GQ blastocysts, whereas the intermediate and poor groups (Q2 + Q1) represented 74 % of the BQ + AE embryos (Supplementary Fig. 3).

## Discussion

As the last point that can be identified and evaluated in an IVF laboratory is the blastocyst formation and quality, a laboratory aiming for best practice must first assess accurately the development potential of a viable cleavage-stage embryo through measurable objective criteria. Attempts to appraise embryo viability based on their metabolism with glucose uptake measurements, or their respiration with oxygen consumption rates, are very promising, but unfortunately not currently available in clinical practice [24, 25]. Therefore, time-lapse technology has become the innovative option in routine embryology laboratories. High-resolution pictures of embryos obtained from fertilization up to the blastocyst stage have offered insightful detail and high precision of mitotic divisions that could not be monitored before.

Models proposed for implementing this new information into a clinical setting brought great enthusiasm in the field and revealed that timings did indeed matter in the prediction of whether an embryo would develop into a blastocyst or implant [15, 17, 20]. Most importantly, the study by Meseguer [17] showed that using quartiles to approach this data set generated by the timed developments coming from the numerous annotations was a sound solution. However, the proposed models could not be widely reproduced and thus be used in a clinical setting where new ways of predicting blastocyst formation and implantation are continuously sought [23].

Time points indicating precise embryo cleavages were shown to be affected by ovarian stimulation protocols, culture media (sequential or single step) and culture conditions (e.g. reduced oxygen) [26–28]. Therefore, in a prediction model that aspires to be widely used, these precise time values or absolute time points cannot be used alone, or in other words cannot be standardized for general applicability across different laboratories with diverse settings. Also, simulating embryo development with absolute time points may not be correct in reflecting the plasticity of embryos.

Furthermore, another time-lapse system introduced recently, Eeva™, is mainly based on time intervals, but the full scoring algorithm is not publically available [15, 22]. For our own data, timings and intervals of early cleavages (until the 4-cell stage) are not sufficient to predict blastocyst formation and quality with high specificity and sensitivity (Table 2; t3-t2, AUC: 0.546; t4-t3, AUC: 0.594).

However, ratios of cleavage timings (CS2-8, CS4-8) and a time interval (t8-t5) we propose here offer the chance of evaluating kinetic data with relative equations that reflect the synchronicity of cell cycles. Therefore, by translating the viability of an embryo into equations that incorporate the cleavage synchronicity observed in the first 3 days of development, CS2-8 has been determined as the most accurate criterion for blastocyst formation and quality prediction (AUC: 0.786). This equation is based on the fact that a human embryo synchronously divides into two cells, remains briefly at the 3-cell stage, and divides again into the longer 4-cell stage. The 5-6-7-cell stages are very rapid compared to the duration of the 4-cell stage. Thus, the equation CS2-8 = ((t3-t2) + (t5-t4)) / (t8-t2) yields the total time ratio of cell evenness until the 8-cell stage. Similarly, the cleavage synchronicity from 4 to 8 cells, CS4-8 = (t8-t5) / (t8-t4), expresses the duration of the 5-6-7-cell stages when compared to the interval from 4 to 8 cells.

In order to identify robust standards that can be readily applicable in a clinical setting, routinely treating many couples of various ages with a wide range of infertility causes, the sole inclusion criterion of the study was to have embryos cultured until day 5. However, the AUCs of the above mentioned formulas differ only within a very small range (≤0.05) at varying patient ages, showing that once the embryo reached the 8-cell stage the expressions described herein are robust enough to predict blastocyst formation and quality (Data not shown). The three relative timings, CS2-8, CS4-8 and t8-t5, can be applied in an IVF setting to rank day 3 embryos with a high probability of becoming TQ + GQ blastocysts without the need to extend the culture of fragile embryos outside the uterine cavity for two more days. Although controversial reports are available listing preterm deliveries, postnatal effects and epigenetic modifications as long-term risks of blastocyst culture, extended embryo culture in laboratory conditions has been shown to allow the selection of embryos that were morphologically similar on day 3 [29, 30]. Kinetic selection of day 3 embryos will certainly improve the outcome of cleavage-stage embryo transfers, which is currently inferior to blastocyst transfer [6]. Moreover, vitrifying kinetically selected embryos on day 3 may reduce manipulations, since blastocyst collapsing is usually performed either with a laser beam or with an ICSI pipette to remove the fluid filling the blastocoel cavity.

Most importantly, CS2-8, CS4-8 and/ or t8-t5 may also help embryologists in the decision to predict the number of TQ + GQ or usable blastocysts, and thus whether to prolong embryo culture until day 5 and improve the decision basis for blastocyst transfer. The relative equations proposed herein will also allow the development of biopsy strategies for PGS and PGD cases by predicting blastocysts that will be available for trophectoderm biopsy. It may also be applied in combination with the risk model developed to rank and sub-select for single embryo transfer [31, 32].

CS2-8 correlated positively with Gardner’s blastocyst morphology criteria, which shows the high predictivity of this equation: 79.2 % of the TQ + GQ blastocysts (Q4) and 86.2 % of the BQ + AE embryos (Q1) were both correctly classified into two quartiles (*p* < 0.0001). Also, the TQ + GQ ratio of embryos located in the highest quartile was found to be nearly six times higher than the lowest quartile (79.2 vs. 13.8 %, respectively).

Morphological observations are prone to intra and interpersonal variations, especially for day 4 and day 5, whereas cleavage stage embryos are more easily assessed, since evaluation of the number and evenness of blastomeres and the degree of fragmentation are more basic and directly measurable. Besides, annotating the time of embryo cleavages by forwarding and rewinding short videos is simpler and generates cleaner data set with less subjectivity as a basis for predictive equations, such as the ones described here.

In this study, among the tested variables, CS4-8 and t8-t5 were also identified as having a good classification power, close to CS2-8, for blastocyst formation and quality (0.776 and 0.778, respectively). We believe that regarding each laboratory’s routine, these two criteria may also give additional insight to the information provided by CS2-8 when predicting the embryo’s viability. However, none of the absolute cell cleavage timings were characterized as being good predictors of blastocyst formation and quality. We suggest that since time intervals and time ratios are not directly related with absolute cleavage timings but rather with the relationship between them, the validity of such equations is more likely to be transferrable between laboratories.

Moreover, they result in a gradient like distribution of embryos that may better fit in the daily routine to prioritize, but most importantly, to rank embryos. Also, as previously reported, defining optimal narrow intervals for mitotic cell divisions to obtain a high specificity at the expense of a low sensitivity would result in discarding many viable embryos [33]. Thus, using relative equations such as time ratios (CS2-8, CS4-8) and time intervals (t8-t5) generating a spectrum-like distribution may give the flexibility and adaptability required for each individual embryo’s evaluation, by avoiding impractical hierarchical classifications based on strict cut-off values that result in only a few subgroups in which more than one embryo can be located, further requiring embryologists’ intervention to make the final selection, rendering the use of kinetic parameters less useful.

In conclusion, we present here for the first time, a detailed comparative analysis of kinetic variables focusing on relative time ratios and time intervals. We also propose expressions of cleavage synchronicity and show that they are good predictors of embryos’ developmental potential. Time points that define precise embryo cleavage events may indeed not be generalized to infertile patients with different etiologies, and may depend on the conditions applied in ART units. However, using time intervals and time ratios based on selected cleavage cycles that define synchronicity of embryos have in this retrospective cohort study allowed an individualized analysis producing a high predictivity of blastocyst formation and quality. The proposed relative equations and intervals need to be further tested in regard to implantation potential and live birth prediction.

## Electronic supplementary material

Below is the link to the electronic supplementary material.

ESM 1 (DOC 325 kb)
