# Supplementary material for: Relative kinetic expressions defining cleavage synchronicity are better predictors of blastocyst formation and quality than absolute time points
Source: J Assist Reprod Genet. 2014 Nov 5;32(1):27–35. doi: 10.1007/s10815-014-0341-x (PMC4294863; doi:10.1007/s10815-014-0341-x)

**Supplementary Table 1** Demographics of patients included in the study

| **n = 648 cycles (626 patients)** | **Mean ± SD** |
| --- | --- |
| Female age | 32.93 ± 5.31 |
| BMI (kg/m^2^) | 24.48 ± 4.01 |
| Gonadotrophin total dose (IU) | 1966 ± 890 |
| Previous IVF cycles | 2.24 ± 2.35 |
| Number of retrieved oocytes | 10.62 ± 5.21 |
| Number of metaphase II oocytes | 7.95 ± 3.67 |
| Number of zygotes | 6.90 ± 2.85 |
| Number of transferred embryos | 1.55 ± 0.52 |

**Supplementary Table 2** Quartile range limits and TQ+GQ blastocysts distribution among quartiles

|  | **Q1*** | **TQ+GQ%** | **Q2*** | **TQ+GQ%** | **Q3*** | **TQ+GQ%** | **Q4*** | **TQ+GQ %** |
| --- | --- | --- | --- | --- | --- | --- | --- | --- |
| **t2** | <24.47 | 65.8 | 24.47-26.56 | 61.4 | 26.56-29.07 | 57.5 | >29.07 | 34.8 |
| **t3** | <34.53 | 45.9 | 34.53-37.36 | 66.4 | 37.36-40.17 | 61.1 | >40.17 | 46.1 |
| **t4** | <36.10 | 60.6 | 36.10-38.71 | 64.2 | 38.71-41.8 | 57.5 | >41.8 | 37.2 |
| **t5** | <45.69 | 36.2 | 45.69-49.96 | 66.0 | 49.96-54.25 | 69.4 | >54.25 | 48.0 |
| **t6** | <48.37 | 53.9 | 48.37-52.28 | 64.9 | 52.28-56.90 | 64.2 | >56.90 | 36.6 |
| **t7** | <50.66 | 64.1 | 50.66-54.72 | 66.3 | 54.72-60.16 | 59.2 | >60.16 | 29.9 |
| **t8** | <52.69 | 69.4 | 52.69-57.6 | 68.2 | 57.6-64.69 | 54.7 | >64.69 | 27.2 |
| **t3-t2** | <10.17 | 36.8 | 10.17-11.26 | 70.8 | 11.26-12.27 | 63.7 | >12.27 | 48.1 |
| **t4-t3** | =0.00 | 52.5 | 0.00-0.67 | 70.9 | 0.67-1.66 | 61.8 | >1.66 | 34.1 |
| **t5-t3** | <11.36 | 36.3 | 11.36-13.01 | 69.6 | 13.01-14.76 | 67.0 | >14.76 | 46.5 |
| **t5-t4** | <9.51 | 22.4 | 9.51-12.00 | 69.6 | 12.00-3.76 | 73.9 | >3.76 | 53.9 |
| **t8-t5** | <3.01 | 80.8 | 3.01-6.33 | 71.0 | 6.33-14.95 | 47.3 | >14.95 | 20.4 |
| **t8-t2** | <26.86 | 66.5 | 26.86-30.43 | 66.7 | 30.43-36.55 | 55.0 | >36.55 | 31.3 |
| **CS2-8** | <0.51 | 13.8 | 0.51-0.76 | 53.6 | 0.76-0.86 | 72.9 | >0.86 | 79.2 |
| **CS2-4** | <0.01 | 56.2 | 0.01-0.05 | 70.4 | 0.05- 0.12 | 64.0 | >0.12 | 33.0 |
| **CS4-8** | <0.19 | 79.7 | 0.19-0.33 | 69.7 | 0.33-0.61 | 54.8 | >0.61 | 15.3 |
| **DR** | <0.73 | 30.0 | 0.73-0.84 | 70.8 | 0.84-0.94 | 74.0 | >0.94 | 49.2 |

*hours

**Supplementary Figure 1** Schematic representation of the cleavage synchronicity from 2 to 8 cells: CS2-8 = ((t3-t2) + (t5-t4)) / (t8-t2). **a** The diagram represents a synchronously dividing embryo spending most of its time in the 2- and 4-cell stages until reaching the 8-cell stage. **b** The diagram represents an asynchronously dividing embryo with very long time periods for 3- 5- 6- and 7-cell stages.


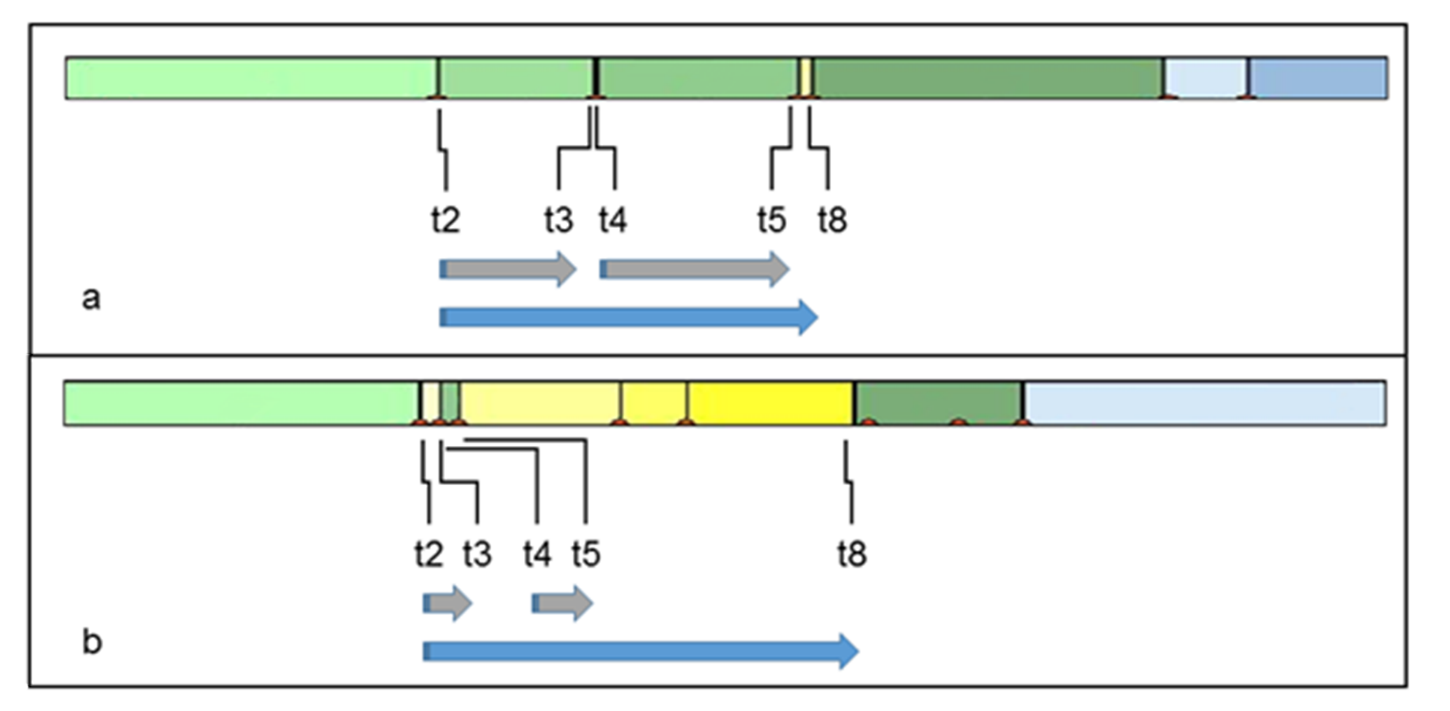


**Supplementary Figure 2** Flow chart of the study


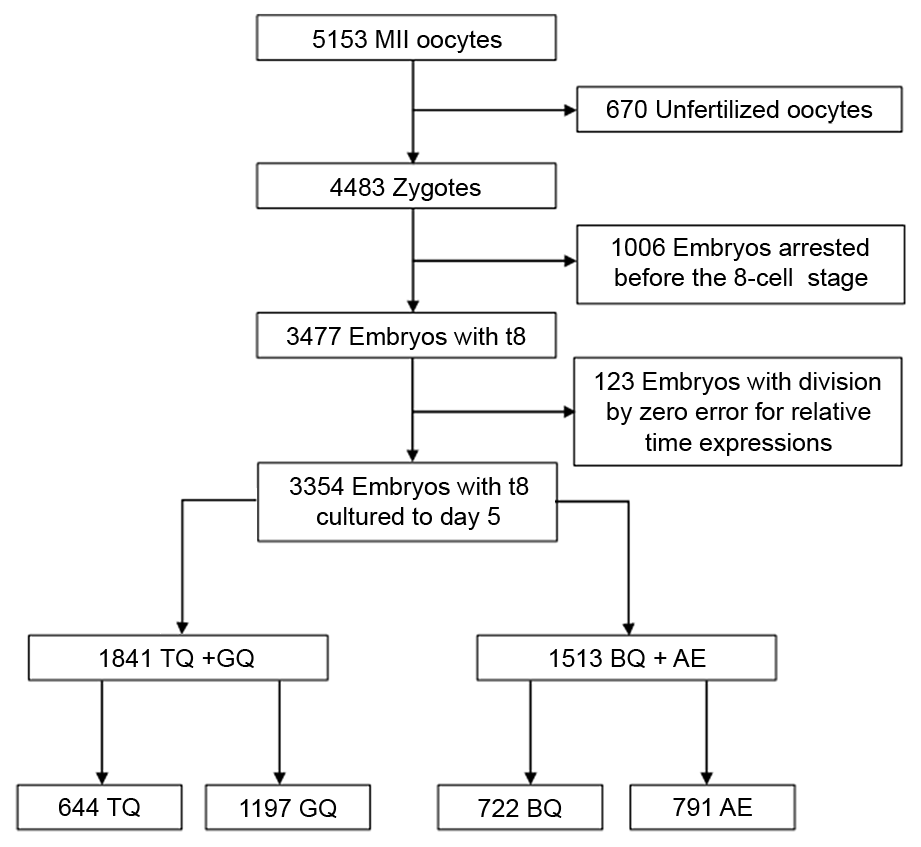


**Supplementary Figure 3** Comparison of the embryo’s final morphology on day 5 with the CS2-8 score given on day 3


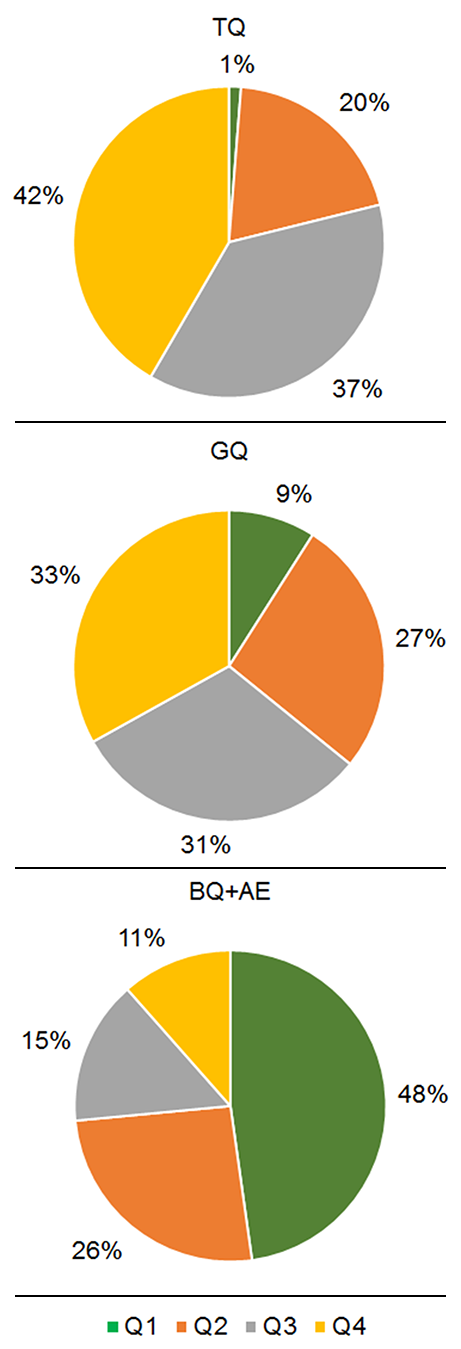

Supplement: Supplementary file 1 — (DOC 325 kb) [file 10815_2014_341_MOESM1_ESM.doc]
